# Fibronectin orchestrates thrombosis and hemostasis

**DOI:** 10.18632/oncotarget.5097

**Published:** 2015-08-03

**Authors:** Yiming Wang, Naadiya Carrim, Heyu Ni

**Affiliations:** Toronto Platelet Immunobiology Group; Department of Laboratory Medicine, Keenan Research Centre for Biomedical Science, St. Michael's Hospital; Canadian Blood Services; Department of Laboratory Medicine and Pathobiology; Department of Physiology; and Department of Medicine, University of Toronto, Toronto, ON, Canada

**Keywords:** fibronectin, thrombosis, hemostasis, platelet, fibrin

Fibronectin (Fn) is an essential extracellular matrix protein involved in cell adhesion, migration, differentiation and proliferation. Fn is required for embryogenesis, and is intricately involved in malignant transformation, angiogenesis, inflammation, fibrosis, wound healing, thrombosis and hemostasis [1]. Fn is a dimer consisting of two 250 kDa subunits. Alternative splicing of Fn pre-mRNA at extra domain (ED)-A, ED-B, and variable region results in up to 20 Fn variants in humans, which are categorized into two major groups, cellular fibronectin (cFn) and plasma fibronectin (pFn). cFn contains at least one of the EDs and is expressed by various cell types. In contrast, pFn is secreted specifically by hepatocytes to the blood circulation and excludes both ED-A and ED-B. Since pFn is an abundant protein in the blood plasma and contains integrin binding motif Arg-Gly-Asp (RGD) as well as N-terminal fibrin and collagen binding sites, it has long been suspected to play a role in thrombosis and hemostasis [2].

Thrombosis is the pathological occlusion or stenosis of blood vessels by platelets and fibrin clots, which often results in heart attack, stroke, and deep vein thrombosis (DVT), the leading causes of mortality and morbidity worldwide [3]. Cancer associated thrombosis is also one of the leading causes of mortality in cancer patients. Recent advances in treatment and prevention of thrombotic diseases have greatly improved patient survival and quality of life. However, many of these therapeutic modalities risk impairing hemostasis (i.e., the physiological process to stop bleeding), leading to life-threatening hemorrhage. On the other hand, therapies to control bleeding can sometimes lead to thrombosis. It is therefore crucial to study the factors involved in the regulation of thrombosis/hemostasis.

In a recent report, we demonstrated that pFn is a vital factor in keeping the balance between thrombosis and hemostasis [4]. We found that pFn is crucial for hemostasis and survival of mice deficient in fibrinogen (a key coagulation factor and the precursor of fibrin) or treated with anticoagulants. Conventional theory states that platelet accumulation is the first wave of hemostatic response to vascular injury. Using our state-of-the-art intravital microscopy models, we found, surprisingly, pFn deposits at the site of injury to support hemostasis even prior to platelet accumulation, and the deposition is independent of platelet, fibrinogen, β3 integrin, or anticoagulants. In addition, we revealed that pFn enhances the mechanical strength of the blood clot by actively incorporating into the fibrin network and markedly increasing the diameter of fibrin fibers. Results from previous studies of pFn in blood coagulation were inclusive, because pFn was inevitably contaminated by fibrinogen (hence the name “fibr(in)o(gen)-nectin”). Here, we used pFn purified from fibrinogen^−/−^ mice, and clarified that pFn significantly strengthens the blood clot.

The role of pFn in platelet aggregation has been debated for several decades [2]. Our previous study using pFn^−/−^ mice showed that pFn promotes thrombus growth and stability in vivo [5]. Interestingly, in a mouse model deficient in both fibrinogen and Von Willebrand Factor (VWF), pFn inhibited platelet aggregation in vitro and thrombosis in vivo [6], suggesting a switch of pFn function in the absence of fibrinogen and/or VWF. In our recent study [4], we found that the fibrin (a product of fibrinogen) is required for pFn to support platelet aggregation and thrombosis. In the absence of fibrin, pFn inhibits thrombosis. In a growing hemostatic plug, fibrin is usually formed at the bottom of the thrombi close to the vessel wall, while at the apical surface of the thrombi, fibrin is almost undetectable. Through covalent linkage with fibrin (forming the pFn-fibrin complex), pFn plays important dual roles by promoting platelet accumulation on the vessel wall to stop blood loss, while curtailing excessive platelet accumulation to the apical surface of the thrombi and preventing excessive thrombosis and vessel occlusion.

pFn is a major component of transfusion products such as fresh frozen plasma and cryoprecipitate, in which the hemostatic benefit of pFn has not been recognized. Our study suggested that pFn is a key hemostatic agent, especially in fibrinogen deficient population and patients treated with anticoagulants. More importantly, we revealed that fibrin non-associated pFn inhibits thrombus growth and vessel occlusion. These findings have established pFn as a self-limiting regulator that maintains the fine balance between thrombosis and hemostasis.

In our in vivo models, we found that large amount of pFn rapidly deposited at the site of vessel injury. These deposited pFn could serve as a matrix to bind many circulating cells and plasma proteins (i.e., growth factors and angiogenic factors). As pFn is associated with various cancer states and has been proposed as a target for anti-tumor therapy [7], it is tempting to hypothesize that the deposited pFn at the vascular injury site is associated with tumor growth and metastasis. Future studies on the deposition of pFn and associated proteins/cells might reveal novel therapeutic targets for thrombosis as well as cancers.
